# Int-Soft (Generalized) Bi-Ideals of Semigroups

**DOI:** 10.1155/2015/198672

**Published:** 2015-01-29

**Authors:** Young Bae Jun, Seok-Zun Song

**Affiliations:** ^1^Department of Mathematics Education, Gyeongsang National University, Jinju 660-701, Republic of Korea; ^2^Department of Mathematics, Jeju National University, Jeju 690-756, Republic of Korea

## Abstract

The notions of int-soft semigroups and int-soft left (resp., right) ideals in semigroups are studied in the paper by Song et al. (2014). In this paper, further properties and characterizations of int-soft left (right) ideals are studied, and the notion of int-soft (generalized) bi-ideals is introduced. Relations between int-soft generalized bi-ideals and int-soft semigroups are discussed, and characterizations of (int-soft) generalized bi-ideals and int-soft bi-ideals are considered. Given a soft set *(α;S)* over *U*, int-soft (generalized) bi-ideals generated by *(α;S)* are established.

## 1. Introduction

As a new mathematical tool for dealing with uncertainties, the notion of soft sets is introduced by Molodtsov [1]. The author pointed out several directions for the applications of soft sets. At present, works on the soft set theory are progressing rapidly. Maji et al. [2] described the application of soft set theory to a decision making problem. Maji et al. [3] also studied several operations on the theory of soft sets. Çağman and Enginoglu [4] introduced fuzzy parameterized (FP) soft sets and their related properties. They proposed a decision making method based on FP-soft set theory and provided an example which shows that the method can be successfully applied to the problems that contain uncertainties. Feng [5] considered the application of soft rough approximations in multicriteria group decision making problems. Aktaş and Çağman [6] studied the basic concepts of soft set theory and compared soft sets to fuzzy and rough sets, providing examples to clarify their differences. In general, it is well known that the soft set theory is a good mathematical model to deal with uncertainty. Nevertheless, it is also a new notion worth applying to abstract algebraic structures. So, we can provide the possibility of a new direction of soft sets based on abstract algebraic structures in dealing with uncertainty. In fact, in the aspect of algebraic structures, the soft set theory has been applied to rings, fields, and modules (see [7, 8]), groups (see [6]), semirings (see [9]), (ordered) semigroups (see [10, 11]), and hypervector spaces (see [12]). Song et al. [11] introduced the notion of int-soft semigroups and int-soft left (resp., right) ideals and investigated several properties.

Our aim in this paper is to apply the soft sets to one of abstract algebraic structures, the so-called semigroup. So, we take a semigroup as the parameter set for combining soft sets with semigroups. This paper is a continuation of [11]. We first study further properties and characterizations of int-soft left (right) ideals. We introduce the notion of int-soft (generalized) bi-ideals and provide relations between int-soft generalized bi-ideals and int-soft semigroups. We discuss characterizations of (int-soft) generalized bi-ideals and int-soft bi-ideals. Given a soft set (*α*, *S*) over *U*, we establish int-soft (generalized) bi-ideals generated by (*α*, *S*).

## 2. Preliminaries

Let *S* be a semigroup. Let *A* and *B* be subsets of *S*. Then the multiplication of *A* and *B* is defined as follows:

(1) $$\begin{array}{c} AB=\left\{ab\in S\;|\;a\in A,b\in B\right\}. \end{array}$$

A semigroup *S* is said to be regular if for every *x* ∈ *S* there exists *a* ∈ *S* such that *xax* = *x*.

A semigroup *S* is said to be left (resp., right) zero if *xy* = *x* (resp., *xy* = *y*) for all *x*, *y* ∈ *S*.

A semigroup *S* is said to be left (right) simple if it contains no proper left (right) ideal.

A semigroup *S* is said to be simple if it contains no proper two-sided ideal.

A nonempty subset *A* of *S* is called

- a subsemigroup of *S* if *AA*⊆*A*, that is, *ab* ∈ *A* for all *a*, *b* ∈ *A*,
- a left (resp., right) ideal of *S* if *SA*⊆*A* (resp., *AS*⊆*A*), that is, *xa* ∈ *A* (resp., *ax* ∈ *A*) for all *x* ∈ *S* and *a* ∈ *A*,
- a two-sided ideal of *S* if it is both a left and a right ideal of *S*,
- a generalized bi-ideal of *S* if *ASA*⊆*A*,
- a bi-ideal of *S* if it is both a semigroup and a generalized bi-ideal of *S*.

A soft set theory is introduced by Molodtsov [1], and Çağman and Enginoğlu [13] provided new definitions and various results on soft set theory.

In what follows, let *U* be an initial universe set and *E* be a set of parameters. Let *P*(*U*) denote the power set of *U* and *A*, *B*, *C*,…, ⊆*E*.

**Definition 1 (see [1, 13]).**

A soft set (*α*, *A*) over *U* is defined to be the set of ordered pairs

(2) $$\begin{array}{c} \left(\alpha ,A\right)≔\left\{\left(x,\alpha \left(x\right)\right):x\in E,\;\alpha \left(x\right)\in P\left(U\right)\right\}, \end{array}$$

where *α* : *E* → *P*(*U*) such that *α*(*x*) = *∅* if *x* ∉ *A*.

The function *α* is called approximate function of the soft set (*α*, *A*). The subscript *A* in the notation *α* indicates that *α* is the approximate function of (*α*, *A*).

For a soft set (*α*, *A*) over *U* and a subset *γ* of *U*, the *γ*-inclusive set of (*α*, *A*), denoted by *i*_*A*_(*α*; *γ*), is defined to be the set

(3) $$\begin{array}{c} i_{A}\left(\alpha ;\gamma \right)≔\left\{x\in A\;|\;\gamma \subseteq \alpha \left(x\right)\right\}. \end{array}$$

For any soft sets (*α*, *S*) and (*β*, *S*) over *U*, we define

(4) $$\begin{array}{c} \left(\alpha ,S\right)\;\tilde{\subseteq }\;\left(\beta ,S\right)\;\text{if}\;\;\alpha \left(x\right)\subseteq \beta \left(x\right)\;\;\forall x\in S. \end{array}$$

The soft union of (*α*, *S*) and (*β*, *S*) is defined to be the soft set $\left(\alpha \;\tilde{\cup }\;\beta ,S\right)$ over *U* in which $\alpha \;\tilde{\cup }\;\beta$ is defined by

(5) $$\begin{array}{c} \left(\alpha \;\tilde{\cup }\;\beta \right)\left(x\right)=\alpha \left(x\right)\cup \beta \left(x\right)\;\forall x\in S. \end{array}$$

The soft intersection of (*α*, *S*) and (*β*, *S*) is defined to be the soft set $\left(\alpha \;\tilde{\cap }\;\beta ,S\right)$ over *U* in which $\alpha \;\tilde{\cap }\;\beta$ is defined by

(6) $$\begin{array}{c} \left(\alpha \;\tilde{\cap }\;\beta \right)\left(x\right)=\alpha \left(x\right)\cap \beta \left(x\right)\;\forall x\in S. \end{array}$$

The int-soft product of (*α*, *S*) and (*β*, *S*) is defined to be the soft set $\left(\alpha \tilde{\circ }\beta ,S\right)$ over *U* in which $\alpha \;\tilde{\circ }\;\beta$ is a mapping from *S* to *P*(*U*) given by

(7) (α ∘~ β)(x)=⋃x=yzα(y)∩β(z)if  ∃y,z∈S  such  that  x=yz∅otherwise.

## 3. Int-Soft Ideals

In what follows, we take *E* = *S*, as a set of parameters, which is a semigroup unless otherwise specified.

**Definition 2 (see [11]).**

A soft set (*α*, *S*) over *U* is called an int-soft semigroup over *U* if it satisfies

(8) $$\begin{array}{c} \;\left(\forall x,y\in S\right)\;\left(\alpha \left(x\right)\cap \alpha \left(y\right)\subseteq \alpha \left(xy\right)\right). \end{array}$$

**Definition 3 (see [11]).**

A soft set (*α*, *S*) over *U* is called an int-soft left (resp., right) ideal over *U* if it satisfies

(9) $$\begin{array}{c} \left(\forall x,y\in S\right)\;\left(\alpha \left(xy\right)⊇\alpha \left(y\right)\left(\text{resp}.,\alpha \left(xy\right)⊇\alpha \left(x\right)\right)\right). \end{array}$$

If a soft set (*α*, *S*) over *U* is both an int-soft left ideal and an int-soft right ideal over *U*, we say that (*α*, *S*) is an int-soft two-sided ideal over *U*.

Obviously, every int-soft (resp., right) ideal over *U* is an int-soft semigroup over *U*. But the converse is not true in general (see [11]).

**Proposition 4 .**

Let (*α*, *S*) be an int-soft left ideal over *U*. If *G* is a left zero subsemigroup of *S*, then the restriction of (*α*, *S*) to *G* is constant; that is, *α*(*x*) = *α*(*y*) for all *x*, *y* ∈ *G*.

**Proof**

Let *x*, *y* ∈ *G*. Then *xy* = *x* and *yx* = *y*. Thus

(10) $$\begin{array}{c} \alpha \left(x\right)=\alpha \left(xy\right)⊇\alpha \left(y\right)=\alpha \left(yx\right)⊇\alpha \left(x\right), \end{array}$$

and so *α*(*x*) = *α*(*y*) for all *x*, *y* ∈ *G*.

Similarly, we have the following proposition.

**Proposition 5 .**

Let (*α*, *S*) be an int-soft right ideal over *U*. If *G* is a right zero subsemigroup of *S*, then the restriction of (*α*, *S*) to *G* is constant; that is, *α*(*x*) = *α*(*y*) for all *x*, *y* ∈ *G*.

**Proposition 6 .**

Let (*α*, *S*) be an int-soft left ideal over *U*. If the set of all idempotent elements of *S* forms a left zero subsemigroup of *S*, then *α*(*x*) = *α*(*y*) for all idempotent elements *x* and *y* of *S*.

**Proof**

Assume that the set

(11) $$\begin{array}{c} I\left(S\right)≔\left\{x\in S\;|\;x\;\;\text{is}\;\;\text{an}\;\;\text{idempotent}\;\;\text{element}\;\;\text{of}\;\;S\right\} \end{array}$$

is a left zero subsemigroup of *S*. For any *u*, *v* ∈ *I*(*S*), we have *uv* = *u* and *vu* = *v*. Hence

(12) $$\begin{array}{c} \alpha \left(u\right)=\alpha \left(uv\right)⊇\alpha \left(v\right)=\alpha \left(vu\right)⊇\alpha \left(u\right) \end{array}$$

and thus *α*(*u*) = *α*(*v*) for all idempotent elements *u* and *v* of *S*.

Similarly, we have the following proposition.

**Proposition 7 .**

Let (*α*, *S*) be an int-soft right ideal over *U*. If the set of all idempotent elements of *S* forms a right zero subsemigroup of *S*, then *α*(*x*) = *α*(*y*) for all idempotent elements *x* and *y* of *S*.

For a nonempty subset *A* of *S* and Φ, Ψ ∈ *P*(*U*) with Φ⊋Ψ, define a map *χ*_*A*_^(Φ,Ψ)^ as follows:

(13) $$\begin{array}{c} \chi_{A}^{\left(\Phi ,\Psi \right)}:S⟶P\left(U\right),\;\;x⟼\left\{\begin{array}{cc} \Phi & \text{if}\;\;x\in A,\\ \Psi & \text{otherwise}. \end{array}\right. \end{array}$$

Then (*χ*_*A*_^(Φ,Ψ)^, *S*) is a soft set over *U*, which is called the (Φ, Ψ)-characteristic soft set. The soft set (*χ*_*S*_^(Φ,Ψ)^, *S*) is called the (Φ, Ψ)-identity soft set over *U*. The (Φ, Ψ)-characteristic soft set with Φ = *U* and Ψ = *∅* is called the characteristic soft set and is denoted by (*χ*_*A*_, *S*). The (Φ, Ψ)-identity soft set with Φ = *U* and Ψ = *∅* is called the identity soft set and is denoted by (*χ*_*S*_, *S*).

**Lemma 8 .**

Let (*χ*_*A*_^(Φ,Ψ)^, *S*) and (*χ*_*B*_^(Φ,Ψ)^, *S*) be (Φ, Ψ)-characteristic soft sets over *U* where *A* and *B* are nonempty subsets of *S*. Then the following properties hold:

1. $\chi_{A}^{\left(\Phi ,\Psi \right)}\;\tilde{\cap }\;\chi_{B}^{\left(\Phi ,\Psi \right)}=\chi_{A\cap B}^{\left(\Phi ,\Psi \right)}$,
2. $\chi_{A}^{\left(\Phi ,\Psi \right)}\;\tilde{\circ }\;\chi_{B}^{\left(\Phi ,\Psi \right)}=\chi_{AB}^{\left(\Phi ,\Psi \right)}$.

**Proof**

(1) Let *x* ∈ *S*. If *x* ∈ *A*∩*B*, then *x* ∈ *A* and *x* ∈ *B*. Thus we have

(14) χAΦ,Ψ ∩~ χBΦ,Ψx=χA(Φ,Ψ)(x)∩χB(Φ,Ψ)(x)=Φ=χA∩B(Φ,Ψ)(x).

If *x* ∉ *A*∩*B*, then *x* ∉ *A* or *x* ∉ *B*. Hence we have

(15) χAΦ,Ψ ∩~ χBΦ,Ψx=χA(Φ,Ψ)(x)∩χB(Φ,Ψ)(x)=Ψ=χA∩B(Φ,Ψ)(x).

Therefore $\chi_{A}^{\left(\Phi ,\Psi \right)}\;\tilde{\cap }\;\chi_{B}^{\left(\Phi ,\Psi \right)}=\chi_{A\cap B}^{\left(\Phi ,\Psi \right)}$.

(2) For any *x* ∈ *S*, suppose *x* ∈ *AB*. Then there exist *a* ∈ *A* and *b* ∈ *B* such that *x* = *ab*. Thus we have

(16) χAΦ,Ψ ∘~ χBΦ,Ψx=⋃x=yzχA(Φ,Ψ)(y)∩χB(Φ,Ψ)(z)⊇χA(Φ,Ψ)(a)∩χB(Φ,Ψ)(b)=Φ,

and so $\left(\chi_{A}^{\left(\Phi ,\Psi \right)}\;\tilde{\circ }\;\chi_{B}^{\left(\Phi ,\Psi \right)}\right)(x)=\Phi$. Since *x* ∈ *AB*, we get *χ*_*AB*_^(Φ,Ψ)^(*x*) = Φ. Suppose *x* ∉ *AB*. Then *x* ≠ *ab* for all *a* ∈ *A* and *b* ∈ *B*. If *x* = *yz* for some *y*, *z* ∈ *S*, then *y* ∉ *A* or *z* ∉ *B*. Hence

(17) χAΦ,Ψ ∘~ χBΦ,Ψx=⋃x=yzχA(Φ,Ψ)(y)∩χB(Φ,Ψ)(z)=Ψ=χAB(Φ,Ψ)(x).

If *x* ≠ *yz* for all *x*, *y* ∈ *S*, then

(18) $$\begin{array}{c} \left(\chi_{A}^{\left(\Phi ,\Psi \right)}\;\tilde{\circ }\;\chi_{B}^{\left(\Phi ,\Psi \right)}\right)\left(x\right)=\Psi =\chi_{AB}^{\left(\Phi ,\Psi \right)}\left(x\right). \end{array}$$

In any case, we have $\chi_{A}^{\left(\Phi ,\Psi \right)}\;\tilde{\circ }\;\chi_{B}^{\left(\Phi ,\Psi \right)}=\chi_{AB}^{\left(\Phi ,\Psi \right)}$.

**Theorem 9 .**

For the (Φ, Ψ)-identity soft set (*χ*_*S*_^(Φ,Ψ)^, *S*), let (*α*, *S*) be a soft set over *U* such that *α*(*x*)⊆Φ for all *x* ∈ *S*. Then the following assertions are equivalent:

1. (*α*, *S*) is an int-soft left ideal over *U*,
2. $\left(\chi_{S}^{\left(\Phi ,\Psi \right)}\;\tilde{\circ }\;\alpha ,S\right)\;\tilde{\subseteq }\;(\alpha ,S)$.

**Proof**

Suppose that (*α*, *S*) is an int-soft left ideal over *U*. Let *x* ∈ *S*. If *x* = *yz* for some *y*, *z* ∈ *S*, then

(19) χSΦ,Ψ ∘~ αx=⋃x=yzχS(Φ,Ψ)(y)∩α(z)⊆⋃x=yzΦ∩α(yz)=α(x).

Otherwise, we have $\left(\chi_{S}^{\left(\Phi ,\Psi \right)}\;\tilde{\circ }\;\alpha \right)(x)=\emptyset \subseteq \alpha (x)$. Therefore $\left(\chi_{S}^{\left(\Phi ,\Psi \right)}\;\tilde{\circ }\;\alpha ,S\right)\;\tilde{\subseteq }\;\left(\alpha ,S\right)$.

Conversely, assume that $\left(\chi_{S}^{\left(\Phi ,\Psi \right)}\;\tilde{\circ }\;\alpha ,S\right)\;\tilde{\subseteq }\;\left(\alpha ,S\right)$. For any *x*, *y* ∈ *S*, we have

(20) αxy⊇χS(Φ,Ψ) ∘~ α(xy)⊇χS(Φ,Ψ)(x)∩α(y)=Φ∩α(y)=α(y).

Hence (*α*, *S*) is an int-soft left ideal over *U*.

Similarly, we have the following theorem.

**Theorem 10 .**

For the (Φ, Ψ)-identity soft set (*χ*_*S*_^(Φ,Ψ)^, *S*), let (*α*, *S*) be a soft set over *U* such that *α*(*x*)⊆Φ for all *x* ∈ *S*. Then the following assertions are equivalent:

1. (*α*, *S*) is an int-soft right ideal over *U*,
2. $\left(\alpha \;\tilde{\circ }\;\chi_{S}^{\left(\Phi ,\Psi \right)},S\right)\;\tilde{\subseteq }\;(\alpha ,S)$.

**Corollary 11 .**

For the (Φ, Ψ)-identity soft set (*χ*_*S*_^(Φ,Ψ)^, *S*), let (*α*, *S*) be a soft set over *U* such that *α*(*x*)⊆Φ for all *x* ∈ *S*. Then the following assertions are equivalent:

1. (*α*, *S*) is an int-soft two-sided ideal over *U*,
2. $\left(\chi_{S}^{\left(\Phi ,\Psi \right)}\;\tilde{\circ }\;\alpha ,S\right)\;\tilde{\subseteq }\;(\alpha ,S)$ and $\left(\alpha \;\tilde{\circ }\;\chi_{S}^{\left(\Phi ,\Psi \right)},S\right)\;\tilde{\subseteq }\;(\alpha ,S)$.

Note that the soft intersection of int-soft left (right, two-sided) ideals over *U* is an int-soft left (right, two-sided) ideal over *U*. In fact, the soft intersection of int-soft left (right, two-sided) ideals containing a soft set (*α*, *S*) over *U* is the smallest int-soft left (right, two-sided) ideal over *U*.

For any soft set (*α*, *S*) over *U*, the smallest int-soft left (right, two-sided) ideal over *U* containing (*α*, *S*) is called the int-soft left (right, two-sided) ideal over *U* generated by (*α*, *S*) and is denoted by (*α*, *S*)_*l*_  ((*α*, *S*)_*r*_, (*α*, *S*)_2_).

**Theorem 12 .**

Let *S* be a monoid with identity *e*. Then (*α*, *S*)_*l*_ = (*β*, *S*), where

(21) $$\begin{array}{c} \beta \left(a\right)=⋃\left\{\alpha \left(y\right)\;|\;a=xy,\;x,y\in S\right\}, \end{array}$$

for all *a* ∈ *S*.

**Proof**

Let *a* ∈ *S*. Since *a* = *ea*, we have

(22) $$\begin{array}{c} \beta \left(a\right)=⋃\left\{\alpha \left(y\right)\;|\;a=xy,\;x,y\in S\right\}⊇\alpha \left(a\right), \end{array}$$

and so $\left(\alpha ,S)\;\tilde{\subseteq }\;(\beta ,S\right)$. For all *x*, *y* ∈ *S*, we have

(23) βxy=⋃α(x2) ∣ xy=x1x2, x1,x2∈S⊇⋃α(z2) ∣ xy=(xz1)z2, y=z1z2,z1,z2∈S⊇⋃α(z2) ∣ y=z1z2, z1,z2∈S=β(y).

Thus (*β*, *S*) is an int-soft left ideal over *U*. Now let (*γ*, *S*) be an int-soft left ideal over *U* such that $\left(\alpha ,S)\;\tilde{\subseteq }\;(\gamma ,S\right)$. Then *α*(*a*)⊆*γ*(*a*) for all *a* ∈ *S* and

(24) βa=⋃α(x2) ∣ a=x1x2, x1,x2∈S⊆⋃γ(x2) ∣ a=x1x2, x1,x2∈S⊆⋃γ(x1x2) ∣ a=x1x2, x1,x2∈S=γ(a).

This implies that $\left(\beta ,S)\;\tilde{\subseteq }\;(\gamma ,S\right)$. Therefore (*α*, *S*)_*l*_ = (*β*, *S*).

Similarly, we have the following theorem.

**Theorem 13 .**

Let *S* be a monoid with identity *e*. Then (*α*, *S*)_*r*_ = (*β*, *S*), where

(25) $$\begin{array}{c} \beta \left(a\right)=⋃\left\{\alpha \left(x\right)\;|\;a=xy,\;x,y\in S\right\}, \end{array}$$

for all *a* ∈ *S*.

## 4. Int-Soft (Generalized) Bi-Ideals

**Definition 14 .**

A soft set (*α*, *S*) over *U* is called an int-soft generalized bi-ideal over *U* if it satisfies

(26) $$\begin{array}{c} \left(\forall x,y\in S\right)\;\left(\alpha \left(xyz\right)⊇\alpha \left(x\right)\cap \alpha \left(z\right)\right). \end{array}$$

We know that any int-soft generalized bi-ideal may not be an int-soft semigroup by the following example.

**Example 15 .**

Let *S* = {*a*, *b*, *c*, *d*} be a semigroup with the following Cayley table:

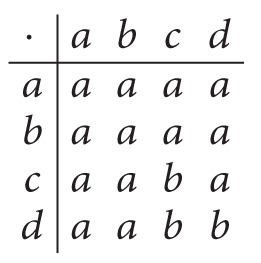
(27)
Let (*α*, *S*) be a soft set over *U* = *Z* defined as follows:

(28) $$\begin{array}{c} \alpha :S⟶P\left(U\right),\;\;x⟼\left\{\begin{array}{cc} 2Z & \text{if}\;x=a,\\ 4N & \text{if}\;x\in \left\{b,d\right\},\\ 4Z & \text{if}\;x=c. \end{array}\right. \end{array}$$

Then (*α*, *S*) is an int-soft generalized bi-ideal over *U* = *Z*. But it is not an int-soft semigroup over *U* = *Z* since *α*(*c*)∩*α*(*c*) = 4*Z*⊈4*N* = *α*(*b*) = *α*(*cc*).

If a soft set (*α*, *S*) over *U* is both an int-soft semigroup and an int-soft generalized bi-ideal over *U*, then we say that (*α*, *S*) is an int-soft bi-ideal over *U*.

We provide a condition for an int-soft generalized bi-ideal to be an int-soft semigroup.

**Theorem 16 .**

In a regular semigroup *S*, every int-soft generalized bi-ideal is an int-soft semigroup, that is, an int-soft bi-ideal.

**Proof**

Let (*α*, *S*) be an int-soft generalized bi-ideal over *U* and let *x* and *y* be any elements of *S*. Then there exists *a* ∈ *S* such that *y* = *yay*, and so

(29) $$\begin{array}{c} \alpha \left(xy\right)=\alpha \left(x\left(yay\right)\right)=\alpha \left(x\left(ya\right)y\right)⊇\alpha \left(x\right)\cap \alpha \left(y\right). \end{array}$$

Therefore (*α*, *S*) is an int-soft semigroup over *U*.

We consider characterizations of an int-soft (generalized) bi-ideal.

**Lemma 17 .**

For any nonempty subset *A* of *S*, the following are equivalent:

1. *A* is a generalized bi-ideal of *S*,
2. the (Φ, Ψ)-characteristic soft set (*χ*_*A*_^(Φ,Ψ)^, *S*) over *U* is an int-soft generalized bi-ideal over *U* for any Φ, Ψ ∈ *P*(*U*) with Φ⊋Ψ.

**Proof**

Assume that *A* is a generalized bi-ideal of *S*. Let Φ, Ψ ∈ *P*(*U*) with Φ⊋Ψ and *x*, *y*, *z* ∈ *S*. If *x*, *z* ∈ *A*, then *χ*_*A*_^(Φ,Ψ)^(*x*) = Φ = *χ*_*A*_^(Φ,Ψ)^(*z*) and *xyz* ∈ *ASA*⊆*A*. Hence

(30) $$\begin{array}{c} \chi_{A}^{\left(\Phi ,\Psi \right)}\left(xyz\right)=\Phi =\chi_{A}^{\left(\Phi ,\Psi \right)}\left(x\right)\cap \chi_{A}^{\left(\Phi ,\Psi \right)}\left(z\right). \end{array}$$

If *x* ∉ *A* or *z* ∉ *A*, then *χ*_*A*_^(Φ,Ψ)^(*x*) = Ψ or *χ*_*A*_^(Φ,Ψ)^(*z*) = Ψ. Hence

(31) $$\begin{array}{c} \chi_{A}^{\left(\Phi ,\Psi \right)}\left(xyz\right)⊇\Psi =\chi_{A}^{\left(\Phi ,\Psi \right)}\left(x\right)\cap \chi_{A}^{\left(\Phi ,\Psi \right)}\left(z\right). \end{array}$$

Therefore (*χ*_*A*_^(Φ,Ψ)^, *S*) is an int-soft generalized bi-ideal over *U* for any Φ, Ψ ∈ *P*(*U*) with Φ⊋Ψ.

Conversely, suppose that the (Φ, Ψ)-characteristic soft set (*χ*_*A*_^(Φ,Ψ)^, *S*) over *U* is an int-soft generalized bi-ideal over *U* for any Φ, Ψ ∈ *P*(*U*) with Φ⊋Ψ. Let *a* be any element of *ASA*. Then *a* = *xyz* for some *x*, *z* ∈ *A* and *y* ∈ *S*. Then

(32) χAΦ,Ψa=χA(Φ,Ψ)(xyz)⊇χA(Φ,Ψ)(x)∩χA(Φ,Ψ)(z)=Φ∩Φ=Φ,

and so *χ*_*A*_^(Φ,Ψ)^(*a*) = Φ. Thus *a* ∈ *A*, which shows that *ASA*⊆*A*. Therefore *A* is a generalized bi-ideal of *S*.

**Theorem 18 .**

A soft set (*α*, *S*) over *U* is an int-soft generalized bi-ideal over *U* if and only if the nonempty *γ*-inclusive set of (*α*, *S*) is a generalized bi-ideal of *S* for all *γ*⊆*U*.

**Proof**

Assume that (*α*, *S*) is an int-soft semigroup over *U*. Let *γ*⊆*U* be such that *i*_*S*_(*α*; *γ*) ≠ *∅*. Let *a* ∈ *S* and *x*, *y* ∈ *i*_*S*_(*α*; *γ*). Then *α*(*x*)⊇*γ* and *α*(*y*)⊇*γ*. It follows from (26) that

(33) $$\begin{array}{c} \alpha \left(xay\right)⊇\alpha \left(x\right)\cap \alpha \left(y\right)⊇\gamma \end{array}$$

and that *xay* ∈ *i*_*S*_(*α*; *γ*). Thus *i*_*S*_(*α*; *γ*) is a generalized bi-ideal of *S*.

Conversely, suppose that the nonempty *γ*-inclusive set of (*α*, *S*) is a generalized bi-ideal of *S* for all *γ*⊆*U*. Let *x*, *y*, *z* ∈ *S* be such that *α*(*x*) = *γ*_*x*_ and *α*(*z*) = *γ*_*z*_. Taking *γ* = *γ*_*x*_∩*γ*_*z*_ implies that *x*, *z* ∈ *i*_*S*_(*α*; *γ*). Hence *xyz* ∈ *i*_*S*_(*α*; *γ*), and so

(34) $$\begin{array}{c} \alpha \left(xyz\right)⊇\gamma =\gamma_{x}\cap \gamma_{z}=\alpha \left(x\right)\cap \alpha \left(z\right). \end{array}$$

Therefore (*α*, *S*) is an int-soft generalized bi-ideal over *U*.

**Lemma 19 (see [11]).**

A soft set (*α*, *S*) over *U* is an int-soft semigroup over *U* if and only if the nonempty *γ*-inclusive set of (*α*, *S*) is a subsemigroup of *S* for all *γ*⊆*U*.

Combining Theorem 18 and Lemma 19, we have the following characterization of an int-soft bi-ideal.

**Theorem 20 .**

A soft set (*α*, *S*) over *U* is an int-soft bi-ideal over *U* if and only if the nonempty *γ*-inclusive set of (*α*, *S*) is a bi-ideal of *S* for all *γ*⊆*U*.

**Theorem 21 .**

For the identity soft set (*χ*_*S*_, *S*) and a soft set (*α*, *S*) over *U*, the following are equivalent:

1. (*α*, *S*) is an int-soft generalized bi-ideal over *U*,
2. $\left(\alpha \;\tilde{\circ }\;\chi_{S}\;\tilde{\circ }\;\alpha ,S\right)\;\tilde{\subseteq }\;\left(\alpha ,S\right)$.

**Proof**

Assume that (*α*, *S*) is an int-soft generalized bi-ideal over *U*. Let *a* be any element of *S*. If $\left(\alpha \;\tilde{\circ }\;\chi_{S}\;\tilde{\circ }\;\alpha \right)(a)=\emptyset$, then it is clear that $\left(\alpha \;\tilde{\circ }\;\chi_{S}\;\tilde{\circ }\;\alpha ,S\right)\;\tilde{\subseteq }\;\left(\alpha ,S\right)$. Otherwise, there exist *x*, *y*, *u*, *v* ∈ *S* such that *a* = *xy* and *x* = *uv*. Since (*α*, *S*) is an int-soft generalized bi-ideal over *U*, it follows from (26) that

(35) $$\begin{array}{c} \alpha \left(uvy\right)⊇\alpha \left(u\right)\cap \alpha \left(y\right) \end{array}$$

and that

(36) α ∘~ χS ∘~ αa=⋃a=xyα∘~χSx∩αy=⋃a=xy⋃x=uvα(u)∩χS(v)∩α(y)=⋃a=xy⋃x=uvα(u)∩U∩α(y)=⋃a=uvyα(u)∩α(y)⊆⋃a=uvyα(uvy)=α(a).

Therefore $\left(\alpha \;\tilde{\circ }\;\chi_{S}\;\tilde{\circ }\;\alpha ,S\right)\;\tilde{\subseteq }\;\left(\alpha ,S\right)$.

Conversely, suppose that $\left(\alpha \;\tilde{\circ }\;\chi_{S}\;\tilde{\circ }\;\alpha ,S\right)\;\tilde{\subseteq }\;\left(\alpha ,S\right)$. For any *x*, *y*, *z* ∈ *S*, let *a* = *xyz*. Then

(37) αxyz=α(a)⊇α ∘~ χS ∘~ α(a)=⋃a=bc(α  ∘~  χS)(b)∩α(c)⊇α ∘~ χS(xy)∩α(z)=⋃xy=uvα(u)∩χS(v)∩α(z)⊇α(x)∩χS(y)∩α(z)=α(x)∩U∩α(z)=αx∩αz.

Therefore (*α*, *S*) is an int-soft generalized bi-ideal over *U*.

**Theorem 22 .**

Let (*α*, *S*) be an int-soft semigroup over *U*. Then (*α*, *S*) is an int-soft bi-ideal over *U* if and only if $\left(\alpha \;\;\tilde{\circ }\;\;\chi_{S}\;\;\tilde{\circ }\;\;\alpha ,S\right)\;\tilde{\subseteq }\;\left(\alpha ,S\right)$, where (*χ*_*S*_, *S*) is the identity soft set over *U*.

**Proof**

It is the same as the proof of Theorem 21.

**Theorem 23 .**

If (*α*, *S*) and (*β*, *S*) are int-soft generalized bi-ideals over *U*, then so is the soft intersection $\left(\alpha \;\tilde{\cap }\;\beta ,S\right)$.

**Proof**

For any *a*, *b*, *x* ∈ *S*, we have

(38) α∩~βaxb=α(axb)∩β(axb)⊇α(a)∩α(b)∩β(a)∩β(b)⊇α(a)∩β(a)∩α(b)∩β(b)=(α∩~β)(a)∩(α ∩~ β)(b).

Thus $\left(\alpha \;\tilde{\cap }\;\beta ,S\right)$ is an int-soft generalized bi-ideals over *U*.

**Theorem 24 .**

If (*α*, *S*) and (*β*, *S*) are int-soft bi-ideals over *U*, then so is the soft intersection $\left(\alpha \;\tilde{\cap }\;\beta ,S\right)$.

**Proof**

It is the same as the proof of Theorem 23.

**Theorem 25 .**

If *S* is a group, then every int-soft generalized bi-ideal is a constant function.

**Proof**

Let *S* be a group with identity *e* and let (*α*, *S*) be an int-soft generalized bi-ideal over *U*. For any *x* ∈ *S*, we have

(39) αx=α(exe)⊇α(e)∩α(e)=α(e)=α(ee)=α((xx−1)(x−1x))=α(x(x−1x−1)x)⊇α(x)∩α(x)=α(x),

and so *α*(*x*) = *α*(*e*). Therefore *α* is a constant function.

For any soft set (*α*, *S*) over *U*, the smallest int-soft (generalized) bi-ideal over *U* containing (*α*, *S*) is called an int-soft (generalized) bi-ideal over *U* generated by (*α*, *S*) and is denoted by (*α*, *S*)_*b*_.

**Theorem 26 .**

Let *S* be a monoid with identity *e* and let (*α*, *S*) be a soft set over *U* such that *α*(*x*)⊆*α*(*e*) for all *x* ∈ *S*. Then (*α*, *S*)_*b*_ = (*β*, *S*), where

(40) $$\begin{array}{c} \beta \left(a\right)=⋃\left\{\alpha \left(x_{1}\right)\cap \alpha \left(x_{3}\right)\;|\;a=x_{1}x_{2}x_{3},\;x_{1},x_{2},x_{3}\in S\right\}, \end{array}$$

for all *a* ∈ *S*.

**Proof**

Let *a* ∈ *S*. Since *a* = *eea* it follows from hypothesis that

(41) βa=⋃α(x1)∩α(x3) ∣ a=x1x2x3, x1,x2,x3∈S⊇α(e)∩α(a)=α(a)

and that $\left(\alpha ,S)\;\tilde{\subseteq }\;(\beta ,S\right)$. For any *x*, *y*, *z* ∈ *S*, we get

(42) β(x)=⋃α(x1)∩α(x3) ∣ x=x1x2x3, x1,x2,x3∈S,βz=⋃αz1∩αz3 ∣ z=z1z2z3, z1,z2,z3∈S,βxyz =⋃αu1∩αu3 ∣ xyz=u1u2u3, u1,u2,u3∈S ⊇⋃α(x1)∩α(z3) ∣ xyz=x1(x2x3yz1z2)z3,‍          x=x1x2x3, z=z1z2z3.

Since

(43) β(x)∩β(z) =⋃(α(x1)∩α(x3))∩(α(z1)∩α(z3)) ∣ ‍    x=x1x2x3,  z=z1z2z3∈S,

we have *β*(*x*)∩*β*(*z*)⊆*β*(*xyz*). Let *y* = *e*. Then *β*(*x*)∩*β*(*z*)⊆*β*(*xz*) for all *x*, *z* ∈ *S*. Hence (*β*, *S*) is an int-soft (generalized) bi-ideal over *U*. Let (*γ*, *S*) be an int-soft (generalized) bi-ideal over *U* such that $\left(\alpha ,S)\;\tilde{\subseteq }\;(\gamma ,S\right)$. For any *a* ∈ *S*, we have

(44) βa=⋃α(x1)∩α(x3) ∣ a=x1x2x3, x1,x2,x3∈S⊆⋃γ(x1)∩γ(x3) ∣ a=x1x2x3, x1,x2,x3∈S⊆⋃γ(x1x2x3) ∣ a=x1x2x3, x1,x2,x3∈S=γ(a),

and so $\left(\beta ,S)\;\tilde{\subseteq }\;(\gamma ,S\right)$. Therefore (*α*, *S*)_*b*_ = (*β*, *S*).
